# The Role of Non-Foraging Nests in Polydomous Wood Ant Colonies

**DOI:** 10.1371/journal.pone.0138321

**Published:** 2015-10-14

**Authors:** Samuel Ellis, Elva J. H. Robinson

**Affiliations:** 1 Department of Biology, University of York, York, YO10 5DD, United Kingdom; 2 York Centre for Complex Systems Analysis, University of York, York, YO10 5DD, United Kingdom; University of Vigo, SPAIN

## Abstract

A colony of red wood ants can inhabit more than one spatially separated nest, in a strategy called polydomy. Some nests within these polydomous colonies have no foraging trails to aphid colonies in the canopy. In this study we identify and investigate the possible roles of non-foraging nests in polydomous colonies of the wood ant *Formica lugubris*. To investigate the role of non-foraging nests we: (i) monitored colonies for three years; (ii) observed the resources being transported between non-foraging nests and the rest of the colony; (iii) measured the amount of extra-nest activity around non-foraging and foraging nests. We used these datasets to investigate the extent to which non-foraging nests within polydomous colonies are acting as: part of the colony expansion process; hunting and scavenging specialists; brood-development specialists; seasonal foragers; or a selfish strategy exploiting the foraging effort of the rest of the colony. We found that, rather than having a specialised role, non-foraging nests are part of the process of colony expansion. Polydomous colonies expand by founding new nests in the area surrounding the existing nests. Nests founded near food begin foraging and become part of the colony; other nests are not founded near food sources and do not initially forage. Some of these non-foraging nests eventually begin foraging; others do not and are abandoned. This is a method of colony growth not available to colonies inhabiting a single nest, and may be an important advantage of the polydomous nesting strategy, allowing the colony to expand into profitable areas.

## Introduction

Foraging is a fundamental part of the life-history strategy of animals. The foraging strategy employed by an animal is dictated by a variety of factors, such as: the type of food resource, the competition for food resources, season and climate [1]. Ants employ a particularly wide range of foraging strategies [2]. This diversity of foraging strategy is likely to be an important factor allowing ants to exploit a wide variety of food sources in a diverse range of habitats [2].

Foraging to honeydew-producing aphids in the canopy provides the majority of the food for red wood ant (*Formica rufa* group) colonies [3]. Red wood ants travel along well-defined trails from their nests to aphid-bearing trees; these trails are composed of thousands of ants foraging to the aphid colonies and can be present in the same position over the course of several years [4–6]. Given the importance of aphids as a food source to the red wood ants, it is surprising that some nests appear not to be foraging. However, previous studies have found that an average of 41% (range: 11%-70%) of the nests within polygynous (multi-queen), polydomous (multi-nest) wood ant colonies have no foraging trails [7]. As workers do not travel to foraging trails originating in other nests, these non-foraging nests have no direct access to honeydew sources, instead they rely on other nests in the colony to provide honeydew [7]. The role of these non-foraging nests in polydomous colonies is unknown. In this study we investigated the possible roles of these apparently ‘non-foraging’ nests.

Polydomous ant colonies inhabit several spatially separated, but socially connected nests. In the case of wood ants, the social connection between nests consists of trails of workers travelling back and forth between the nests of the colony, much like on the foraging trails. Polydomy is a common nesting strategy in ants; it is present in at least 166 ant species from 49 genera [8], including many ecologically dominant species (e.g. *Oecophylla smaragdina*; [9]) and many invasive species (e.g. *Linepithema humile*; [10]). There does not appear to be an ecological or functional niche shared by polydomous species; it may be that the benefits of a polydomous nesting strategy vary from species to species. [8,11]. In red wood ants the main benefit of polydomy has been hypothesised to be to help them exploit dispersed, but spatially and temporally stable, honeydew sources [12]. This is supported by recent results showing that the number of ants on the trails between nests within the colony is related to the difference in the amount of foraging being done by the nests being connected [7]. Specifically, on trails between a non-foraging and a foraging nest the number of ants on the trail (controlled for the size of the nest) is related to the amount of foraging being performed by the foraging nest [7]. This suggests that the structure of the trail network in polydomous colonies is driven by the local redistribution of honeydew between nests. The importance of honeydew exchange in structuring the polydomous colony makes it particularly unexpected that such a high proportion of nests within polydomous colonies appear not to be foraging.

**Fig 1:**
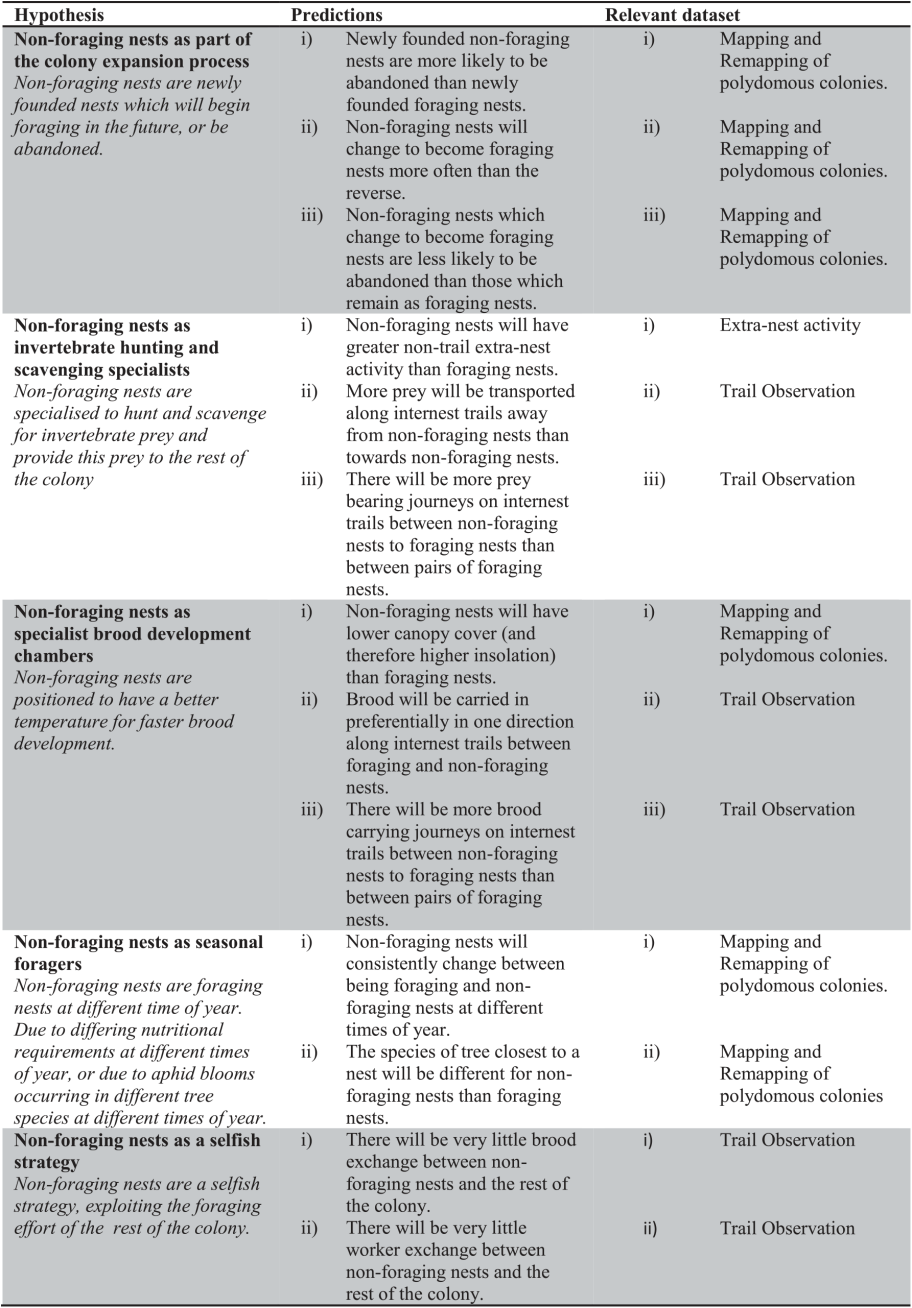
Hypothesised roles of non-foraging nests within polydomous *Formica lugubris* colonies. The predictions, and datasets used to test these predications, resulting from the hypothesis (described in italics) are also listed.

We suggest five possible, non-exclusive, roles that these non-foraging nests may perform in polydomous colonies (Fig 1). (1) Non-foraging nests could be part of the process of *colony expansion*. Polydomous wood ant colonies can expand by founding new nests by the process of budding: during budding a section of workers and queens leave the natal nest on foot and build a new nest nearby, which remains socially connected to the rest of the colony. It may be that non-foraging nests are newly founded nests which will, in the future, begin foraging, or be abandoned. (2) Nests which are apparently non-foraging could act as *arthropod hunting and scavenging specialists*. We define a non-foraging nest as a nest without foraging trails to aphid colonies in the trees. However, wood ants also require protein, provided by hunting and scavenging a variety of invertebrate prey [13–15]. It may be that the non-foraging nests, defined as nests not foraging for honeydew in trees, are actually important as scavengers and hunters of other invertebrate prey. (3) Non-foraging nests could act as *specialised brood development chambers*. In insects, brood temperature is closely linked to development speed, and therefore fitness [16]. External temperature is an important determinant of internal nest temperature in wood ant nests; especially in nests with smaller populations which are less able to create warmth via metabolic heat production [17,18]. It may be that non-foraging nests are in areas with different temperature regimes than foraging nests and can therefore act as specialised brood development chambers for other nests in the colony. (4) Apparently non-foraging nests could appear as a result of *seasonal foraging behaviours*. Aphids may bloom in different species of trees at different times of year [19], and it may therefore be beneficial for a colony to have nests near to trees which will be good for foraging at a different times of year. Nests which appear to be non-foraging at a particular time could, therefore, simply be foraging at other times of year. (5) Finally, non-foraging nests could simply be part of a *selfish strategy*, exploiting the foraging effort of the rest of the colony. A selfish strategy would mean that non-foraging nests are not providing a direct fitness benefit to the rest of the colony.

This study aims to differentiate between these hypothesised roles of non-foraging nests in polydomous wood ant colonies. Several predictions can be made based on each hypothesis (Fig 1) and we collected three empirical datasets to test these predictions: network remapping, trail observations and extra-nest activity counts.

## Methods

### Study species and site

*Formica lugubris* is a member of the ecologically important red wood ant species group (*F*. *rufa* group). The red wood ant group consist of at least six closely related and ecologically similar species [20,21]. The red wood ants are characterised by their large nests constructed of pine needles and leaf litter over subterranean chambers; these nests can be large and contain over a million individuals [7,17]. Members of the group show diversity in nesting strategy both within and between species. This diversity in nesting strategy is associated with a matching flexibility in number of queens in each nest. Polydomous colonies are polygynous (multiple queens per nest) whereas monodomous colonies usually have only one queen per nest [12].

This study took place on a large population (over 900 nests in 0.95 ha^-1^) of *F*. *lugubris* at the Longshaw Estate, Peak District, England (53° 18.55 N, -1° 36.16 W). The area studied is on a west facing slope between 260m and 350m above sea level. All studies were undertaken between late-spring (May) and late summer (September). During this period daytime temperatures range from approximately 10°C to 25°C, with an of average approximately 9 days of rainfall per month [22]. *Formica lugubris* is the only member of the *F*. *rufa* group at this site. The site is a mixture of historic scots pine plantations, deciduous and mixed woodlands and open grassland areas.

**Fig 2:**
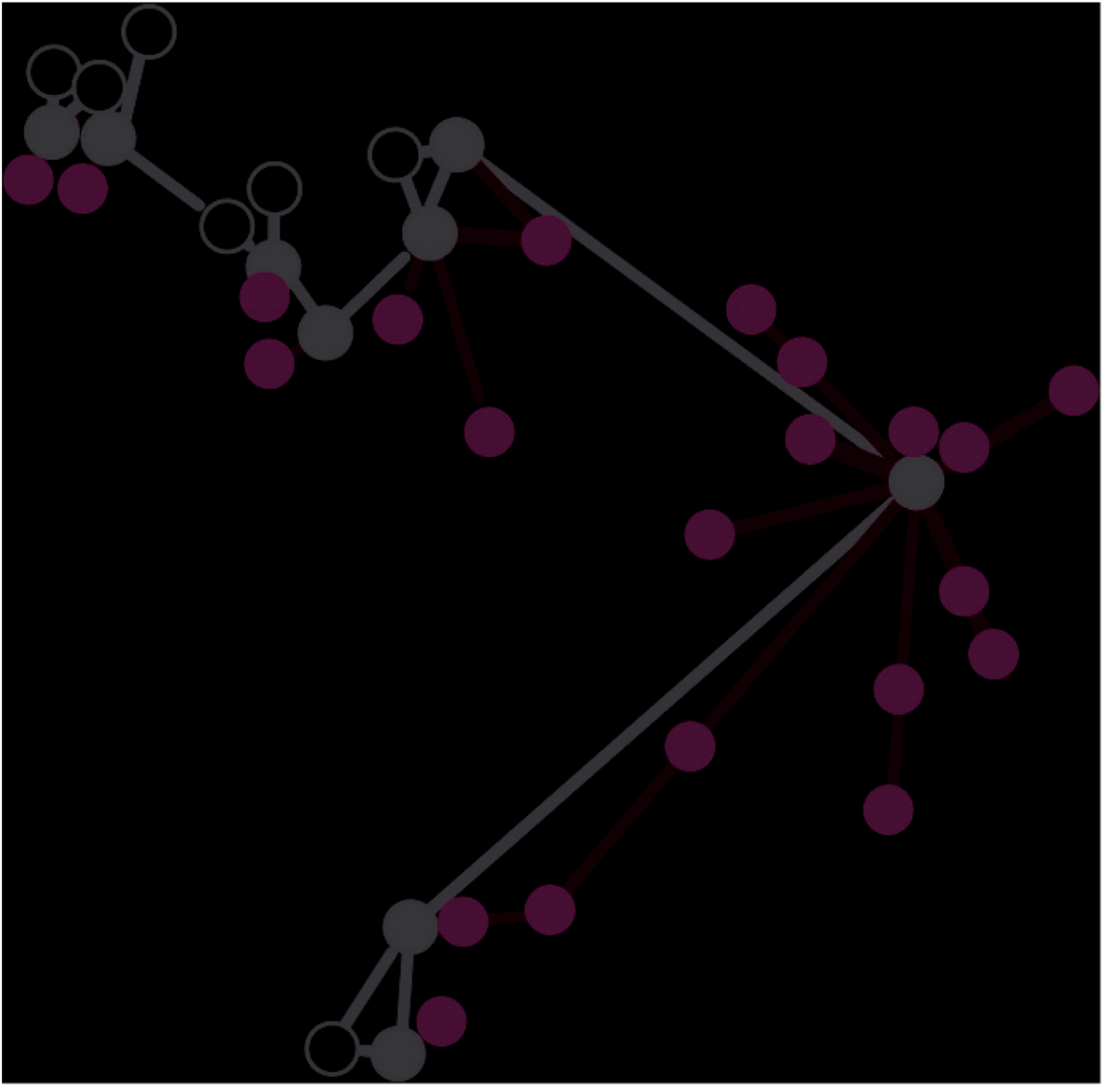
A polydomous *Formica lugubris* colony. Black circles represent foraging nests; open circles show non-foraging nests. Green circles are trees. Black lines are internest trails and green lines are foraging trails. Any nest without foraging trails leading to a tree is defined as non-foraging.

To assess the role of non-foraging nests in polydomous wood ant colonies it is first necessary to define a ‘non-foraging nest’. We define a non-foraging nest as: a nest which is part of a polydomous colony, but has no foraging trails leading to trees (e.g. Fig 2). We follow Ellis et al [7] and define a trail as a route between two points with more than 10 ants within 40 cm. This gives us a functional definition of a polydomous colony based on resource exchange along the trails between nests, rather than based on aggression or relatedness between nests. Preliminary surveys of the site in early-summer (late-May) 2012 and early summer (late-May) 2013 identified a range of polydomous colonies with non-foraging nests which could be used for the investigations.

### Dataset 1: Mapping and remapping colonies

To assess the foundation and survival of nests in polydomous colonies we remapped the same colonies five times over three years. The thirteen largest polydomous colonies from the early-summer 2012 preliminary survey were selected for use in this investigation. These thirteen colonies were then mapped in detail (discussed below) in August 2012, and then remapped in May and late-August 2013, and then again in May and late-August 2014. This gives five time-points for the 13 colonies, three from late-August (hereafter late-summer) and two in May (hereafter spring). The precise timing of the spring remapping depended on when the weather became warm enough for the foraging and internest trails to become active.

In this study, we have defined a polydomous colony as two or more nests connected by trails of ants travelling between them. The trails are above ground and, usually, easy to observe. Similar trails are formed from the nests to the aphid colonies in the trees. During mapping, we recorded the layout of these nests and the trails between them (Fig 2). In addition, we assessed both the populations of the nests in the network and the canopy cover over the nests. Nest population (hereafter nest size) was assessed based on the nest-mound volume, calibrated with a mark-release recapture method [7,23]. At every remapping we estimated canopy cover from digital photos taken vertically 30cm above the highest point of the nest [7]. Photographs were taken using a standard digital camera. We then used image analysis software [24] to calculate the proportion of the area directly above the nest shaded by the canopy [7]. This method of canopy cover measurement has been used in previous studies of red wood ant nests [7,15].

We used these maps to test the predictions associated with the hypothesised roles of non-foraging nests (Fig 1). For example, the foundation of new nests can be inferred by comparing a colony to its previous time-point and observing which nests have appeared. Similarly, nest abandonment can be inferred by examining the colony map from the next time-point and assessing the presence or absence of the nest. As the maps also include foraging trails the foraging or non-foraging status of a nest can be determined by the presence or absence of foraging trails at a particular time-point. This information can also be used to infer changes in foraging status by comparing the same nest at different time-points. We also used the maps to assess the canopy cover over particular nests, and the linear distance (rather than trail distance) to the nearest tree.

### Dataset 2: Trail observation

To understand the role of non-foraging nests it is important to know what resources are being exchanged between non-foraging nests and other nests in the polydomous network. We use ‘resources’ to refer to items being carried by workers between the nests; the resources being carried over the course of the observation periods could be categorised as either: prey, vegetation (nesting material), workers, pupae, larvae, empty pupal casings and queens. It is important to note that this list does not include honeydew, which is transported within the crop of the workers (i.e. internally and therefore not carried); the transport of honeydew between nests is discussed in more detail in [25].

These observations were conducted in July and August 2013. Eight colonies containing both foraging and non-foraging nests were randomly selected from the colonies surveyed in early-summer 2013. Before the beginning of observations the colonies were mapped in detail (see above). One trail between a non-foraging nest and a foraging nest, and one trail between two foraging nests, were randomly selected per colony. The mean length of these trails was 2.81m ± 1.34m (sd).

Observations took place approximately mid-way between the two nests. At the mid-point we designated a 5cm section of trail as the observation window; if an ant carrying a resource traversed the length of this observation window it was considered to be travelling in that direction. Each trail was observed for 30 minutes on 3 consecutive days. Preliminary work showed that neither a longer sampling window (one hour rather than 30 minutes) nor longer term monitoring (for six days rather than three days) had a significant effect on the proportions of resources observed being transported. The two trails per colony were observed in immediate succession in a random order. All observations were done between 10:00 and 17:00 in warm dry weather.

Along a trail between a non-foraging nest and a foraging nest, resources could either be carried towards the non-foraging nest or away from the non-foraging nest. We could then compare the resources being carried towards and away from non-foraging nests. We could also compare the resources being carried between non-foraging nests and foraging nests to those being transported between two foraging nests.

### Dataset 3: Extra-nest activity

The aim of this investigation was to study whether there is a difference in extra-nest activity in the area around non-foraging nests compared to the area around foraging nests. The investigation was conducted at the same time, and used the same eight colonies, as the trail observations (dataset 2, above).

We used counts of activity in a defined area surrounding the nest to assess the extra-nest activity. This method takes into account both the activity-level of extra-nest workers and number of extra-nest workers. Similar methods have been used previously to assess abundance and activity of ants in the area surrounding nests [26]. We used two 15x15 cm squares of cardboard (hereafter: quadrants) placed 40cm from the edge of the focal nest at a randomly selected cardinal direction, to assess the activity in the area surrounding the nests. The squares were always placed 15cm or further from internest and foraging trails. Preliminary observation had shown that these distances were far enough away from the nest to avoid the confounding effects of ants joining, leaving and straying from trail and nests. A second quadrant was placed on the opposite side (180°) of the nest from the first. Quadrants were placed at least 60 minutes before the beginning of the observation to allow the extra-nest workers to acclimatise to them. Each quadrant was observed continuously for 15 minutes and the number of ants passing across the quadrant was recorded. Observations were repeated on three consecutive days. On each day the first quadrant for observation was chosen randomly, and subsequent observations alternated between the two nests being observed in the colony. For analysis the activity in both quadrants were summed and then divided by the worker population of the nest to give a metric for extra-nest activity, given the size of the nest.

Temperature is an important determinant of activity in ants (e.g. [4]). To be able to take account of temperature in our analysis we used a digital thermometer placed 50cm from the nest at the beginning of each observation to take the local temperature accurately, on a short time scale. Another important determinant of extra-nest activity is the number of ants present in the nest. To assess the nest population we used volume of the nests calibrated with a mark-release recapture method based on nest disturbance [7,23].

### Statistical analysis

Statistical analysis of this data was undertaken using Generalised Linear Mixed Effect Models (GLMMs). For dataset 1 we studied the same colony for several time-points, and for datasets 2 and 3 we studied the same colony for several days. GLMMs allow this nesting to be taken into account in the analysis. GLMMs associated with dataset 1 used colony, year and, when appropriate, nest ID as random effects. GLMMs associated with datasets 2 and 3 used colony and day as nested random effects. Additionally, for dataset 3, temperature was also included as a random effect. The fixed effects(s) and response variable were chosen based on the question being asked. Further details of all reported tests are in S1 Table; the superscript number by each reported test refers to the row of the table (S1 Table). All results are based on an Analysis of Deviance (AoD) between the GLMM in question and a null model based on the same variables but without the fixed effect; using this method allows a quantitative assessment of the significance of a particular variable in explaining the modelled data. All analysis was performed in R using the ‘lmer’ and ‘languageR’ packages [27]. Descriptive statistics are reported as mean ± standard error.

### Ethics statement

All datasets were collected at the Longshaw estate. This estate is owned by the National Trust who gave permission for the work to be carried out on their land. *Formica lugubris* is not protected in the UK. None of the data collection involved either sampling or manipulating the ants, and all disruption was kept to a minimum. All methods conformed to the ASAB/ABS ethical guidelines for the use of animals in research [28].

## Results and Discussion

In this study we assessed the extent to which non-foraging nests in polydomous *F*. *lugubris* colonies are: (1) part of the colony expansion process, (2) arthropod hunting and scavenging specialists, (3) specialist brood development chambers, (4) seasonal foragers or (5) a selfish strategy exploiting the foraging effort of the rest of the colony. These hypotheses are not mutually exclusive, as the non-foraging nests in polydomous colonies do not necessarily all have the same role.

### (1a) Non-foraging nests as part of a colony expansion process

#### Predictions

Non-foraging nests could be part of the process of colony expansion in polydomous wood ants, acting as an intermediate phase between nest foundation and the beginning of foraging. The mechanism of colony expansion may be based on established nests budding new nests, some of which happen to be non-foraging. Those which do happen to be non-foraging are retained if they begin foraging and abandoned if they do not. Under this hypothesis rather than non-foraging nests being non-foraging *per se* they have simply not begun foraging.

The hypothesis that new nests are part of the process of colony expansion leads to several predictions (Fig 1). Firstly, we predict that newly founded non-foraging nests are more likely to be abandoned than newly founded foraging nests. Secondly, we expect that non-foraging nests will become foraging nests more often than foraging nests stop foraging and become non-foraging nests. We predict this asymmetrical change because, under the hypothesis that non-foraging nests are an intermediate phase between nest foundation and the beginning of foraging, non-foraging nests at a particular time-point will become foraging nests in the future, if they survive. Thirdly, we predict that non-foraging nests that do change to become foraging nests are less likely to be abandoned than non-foraging nests that have remained as non-foraging nests. This is expected because those which have changed are now fulfilling a role as foraging nests, and are therefore more likely to be retained.

#### Results

**Fig 3:**
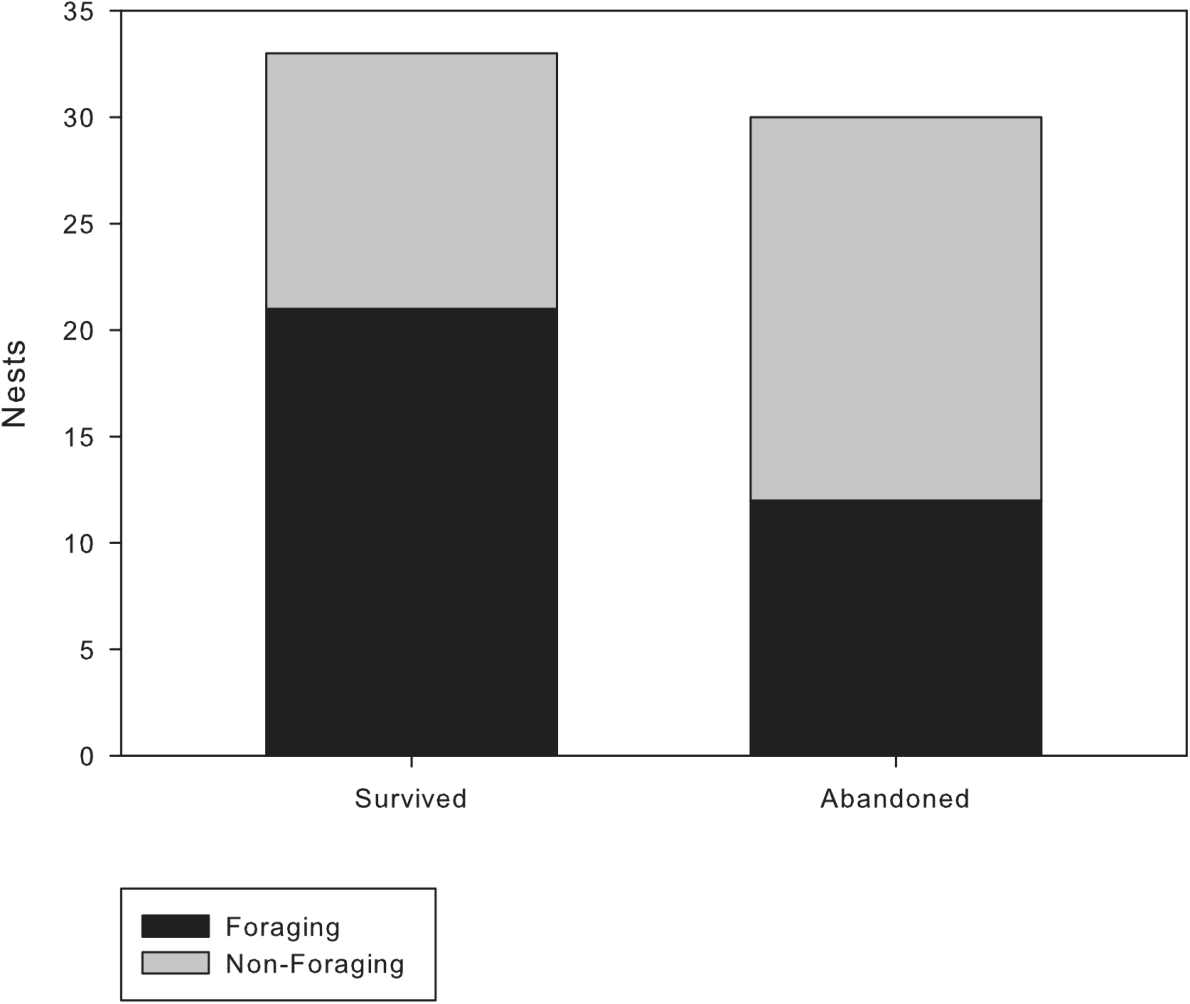
Comparing the survival of newly founded foraging nests (black) and the survival of newly founded non-foraging nests (grey). Significantly more newly-founded non-foraging nests are abandoned before the next observation than newly founded foraging nests (non-foraging: 60% vs. foraging: 36%; AoD^2^, χ^1^ = 5.63, *df* = 1, p = 0.01).

Over the course of the three years of observation we detected the foundation of 91 new nests. Of these newly founded nests 55 were foraging and 36 were non-foraging. Newly founded non-foraging nests are significantly more likely to be abandoned than newly founded foraging nests (non-foraging: 60% vs. foraging: 36%; AoD^1^, χ^2^ = 5.63, *df* = 1, p = 0.01; Fig 3). In general, larger nests are significantly more likely to survive than smaller nests (AoD^2^, χ^2^ = 56.1, *df* = 1, p<0.001). However, even if size is included in the model, foraging status is still a significant determinant of the survival of newly founded nests (AoD^3^, χ^2^ = 5.64, *df* = 1, p = 0.03), whereas size is not (AoD^4^, χ^2^ = 0.01, *df* = 1, p = 0.34).

**Fig 4:**
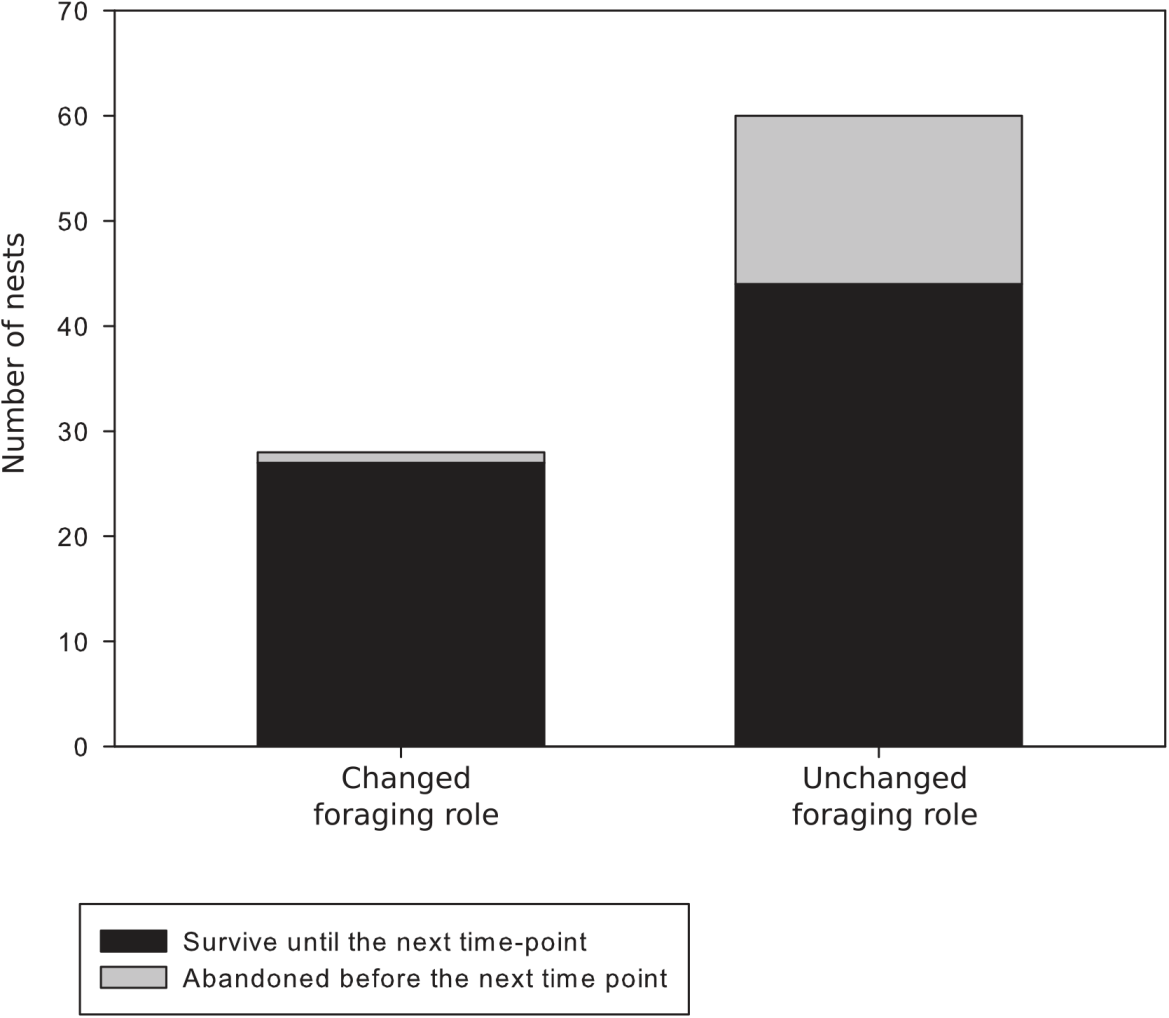
Comparing the survival of nests which change from non-foraging to foraging to those which remain as non-foraging nests (black: nest which survive, grey: nests which are abandoned). Nests which change role are significantly more likely to survive than those that do not (changed, 27/28 survive; unchanged, 44/60 survive; AoD^7^, χ^2^ = 9.5, *df* = 1, p = 0.002).

Of the non-foraging nests which survive between two time-points (e.g. between summer 2012 and spring 2013, or between spring and summer 2014) 36% (36/100) become foraging nests. In contrast, only 14% (32/256) of foraging nests change to become non-foraging nests. There are significantly more changes from non-foraging to foraging than from foraging to non-foraging (AoD^5^, χ^2^ = 13.7, *df* = 1, p<0.001). The non-foraging nests which change to become foraging nests are significantly closer to trees, relative to other nests in the colony, than newly founded nests which remain as non-foraging nests (AoD^6^, χ^2^ = 4.21, *df* = 1, p = 0.04). Of the non-foraging nests that survive between two time-points, those which change to become foraging nests are significantly less likely to be abandoned by the subsequent time-point than those which remain as non-foraging nests (changed, 27/28 survive; unchanged, 44/60 survive; AoD^7^, χ^2^ = 9.5, *df* = 1, p = 0.002; Fig 4).

#### Discussion

Examination of the changes in the polydomous colonies over time shows that non-foraging nests may simply appear as part of the process of colony expansion, rather than having a specific role. Overall, as predicted under the colony expansion hypothesis, newly founded non-foraging nests are more likely to be abandoned than newly founded foraging nests. In addition, non-foraging nests are more likely to change foraging role than foraging nests. Those non-foraging nests which do change foraging role to become foraging nests are both closer to trees and more likely to survive than those which remain as non-foraging nests. Further work is necessary to establish the relationship between survival and foraging, and the factors influencing the survival of a nest in a polydomous colony. This supports the hypothesis that non-foraging nests are part of the process of colony expansion in *F*. *lugubris* because it suggests a mechanism by which polydomous colonies expand: new nests are founded some of which happen to be non-foraging, of these non-foraging nests those which are near to trees become foraging nests in time and those which are further away from trees do not. Those non-foraging nests which have begun foraging are retained whereas those that have not are abandoned. This will result in the observed pattern of a large number of non-foraging nests founded, but only a few that are retained. This process of colony expansion resembles the pruning-based growth patterns found in a variety of biological systems, where a system expands rapidly and then parts in unproductive areas are progressively abandoned (e.g. [29–31]).

### b) The mechanism of nest foundation in polydomous wood ant colonies

#### Predictions

A mechanism of colony expansion based on nests being founded and then abandoned if they are not profitable could function in one of two ways. New nests could be founded in random locations in the area surrounding the colony (random foundation). Conversely, nests could be preferentially founded in areas which may be profitable to the colony, for example close to existing food sources (directed foundation). Both of these mechanisms would result in a similar pattern of non-foraging nests being founded and then either changing to become foraging nests, or being abandoned. Even under the ‘directed’ mechanism just because a nest is founded in an area close to a food sources does not necessarily mean that it will be profitable.

Under a directed process of nest foundation, a location that is very likely to be profitable to found a new nest is on a foraging trail. Founding on a foraging trail will allow the new nest easy access to an already exploited food source. Foundation on existing trails has been previously suggested as a mechanism of nest foundation for wood ant colonies (e.g. [32]). Even if nests are not founded directly on foraging trails it would be beneficial for them to be founded nearer to trees than their natal nest. We therefore expect that, under directed nest foundation, new nests will be founded closer to trees than the existing nests in the colony. In contrast, nests founded at random are expected to be founded, on average, at the same distance from foraging trees as other nests in the colony.

#### Results

We observed the foundation of 91 nests over the course of the three years of observation. Of these, 16 (17.6%) were founded on foraging trails and 19 (20.8%) were founded on internest trails. In total, therefore, 60% of nests were founded in new locations, compared to 40% on existing trails. The newly-founded nests are not significantly closer to foraging trees than other nests in the colony (newly founded 8.48 ± 1.22 m vs. not newly founded 5.95 ± 0.3 m; AoD^8^, χ^2^ = 0.19, df = 1, p = 0.66).

#### Discussion

New nests to do not appear to be founded preferentially in areas that may be beneficial for foraging, rather they appear to be founded in random locations with respect to food sources. This may help explain why the proportion of non-foraging nests in many colonies is so high.

We have found that new nests are founded at random with respect to food sources (hypothesis 1b), but those which happen to be in a location which allows them to begin foraging survive, whereas those which do not begin foraging are abandoned (hypothesis 1a). Therefore, despite not appearing to be directly involved with foraging, non-foraging nests are involved in the honeydew collection process, acting as an intermediate stage before a nest begins foraging. This highlights the importance of the spatially and temporally stable resource of honeydew to red wood ants: even nests which are not foraging to honeydew are part of a mechanism to more efficiently exploit honeydew sources in the environment. Our results also illustrate a possible link between foraging to honeydew and polydomous nesting in the red wood ants. An important benefit of polydomy for red wood ants may be to more efficiently exploit stable food sources in the environment [12]. Founding new nests as non-foraging nests may allow colonies to discover new food sources, or to more efficiently exploit already known food sources; by, for example, allowing multiple nests to be involved in the recruitment of workers to the resource, or by reducing the costs associated with long foraging trails [11]. As this method of exploring the environment is only available to polydomous nests it may provide an important benefit of the polydomous nesting strategy.

### (2) Non-foraging nests as hunting and scavenging specialists

#### Predictions

We predict that, under the hypothesis that non-foraging nests are hunting and scavenging specialists, non-foraging nests will show greater extra-nest activity than expected for their size, as they have more foraging effort invested in hunting and scavenging the area around the nest. If non-foraging nests are providing protein to the rest of the colony it is expected that the net movement of transported prey will be towards foraging nests from the non-foraging nests. Similarly, we predict that, if non-foraging nests are involved in hunting and scavenging, the amount of prey being carried along trails between non-foraging and foraging nests will be higher than on trails between pairs of foraging nests.

#### Results

Non-foraging nests have significantly higher extra-nest activity than foraging nests (AoD^9^, χ^2^ = 19.2, *df* = 1, p<0.001). However, this relationship between extra-nest activity and foraging status is significantly different between colonies (AoD^10^, χ^2^ = 54.7, *df* = 14, p<0.001), suggesting that this difference in extra-nest activity is not a consistent effect.

There is no significant difference in the direction which prey is carried along trails between non-foraging and foraging nests (AoD^11^, χ^2^ = 0.04, *df* = 1, p = 0.84). Similarly, and contrary to prediction of the prey-specialist hypothesis, a significantly higher proportion of ants travelling between foraging nests are carrying prey than those travelling between non-foraging nests and foraging nests (3.3 ±2.2% and 0.6 ±0.2% of journeys respectively; AoD^12^, χ^2^ = 128, *df* = 15, p<0.001).

#### Discussion

Non-foraging nests do not appear to act as sources of hunted and scavenged prey to the rest of the colony. There is higher extra-nest activity in the area surrounding non-foraging nests. However, the low proportion, and lack of consistent direction, of prey bearing journeys on trails between non-foraging nests and foraging nests suggests that non-foraging nests are not hunting and scavenging specialists for the colony.

Our results do not rule out non-foraging nests performing a disproportionate amount of hunting and scavenging but, if they do not appear to supply this excess to the rest of the colony. The higher levels of extra-nest activity around non-foraging nests does not necessarily suggest higher scavenging and hunting effort, it could also be due to searching for other resources such as nest material, or a be a defensive measure. It should also be noted, that our definition of foraging nest based on honeydew collection does not preclude foraging nests from also collecting prey. Indeed, several studies have found that a large proportion of the protein intake of wood ant colonies is provided by hunting and scavenging in the canopy, including on the aphids themselves [3,15,33,34]. In this study we found that more prey is carried along trails between two foraging nests. This could be due to the majority of prey being collected in the canopy.

Overall, it appears unlikely that non-foraging nests are acting as hunting and scavenging specialists, and providing protein to the rest of the colony. There does not appear to be any nest-level division of labour with respect to collection of protein.

### (3) Non-foraging nests as brood development specialists

#### Predictions

Due to the important link between temperature and brood development speed in insects it may be beneficial for polydomous *F*. *lugubris* colonies to place non-foraging ‘brood development’ nests in areas of favourable temperatures (found in seasonally polydomous *Myrmica punctiventris*; [35]). Therefore, under the hypothesis that non-foraging nests are brood development specialists, we predict that non-foraging nests will be in areas with different insolation than foraging nests. In addition, if non-foraging nests are involved in brood rearing we predict greater brood-carrying activity on trails between non-foraging nests and foraging nests. We also predict that the movement of brood along trail these trails between non-foraging and foraging nests will be directional. Depending on the precise brood development role non-foraging nests are fulfilling, this direction could be either towards or away from non-foraging nests. For example, it may be that, due to differing temperature requirements, brood are taken to the non-foraging nest as pupae and taken back to foraging nests as larvae, or *vice versa*. Brood could be also be moved to non-foraging nests in response to some weather conditions, but away from non-foraging nests in other weather conditions. In all these cases, for a specific trail on a specific day, the movement of brood is predicted to be directional. For social insects the main consumers of protein are brood (e.g. [36]). Non-foraging nests acting as brood development specialists might, therefore, be expected to receive a disproportionately higher amount of protein prey than expected for their size, and/or have higher extra-nest activity than foraging nests (Fig 1).

#### Results

We used canopy cover as a proxy for insolation (see methods). We found no significant difference in canopy cover over non-foraging nests compared to foraging nests (20 ± 3% and 25 ± 6% respectively; AoD^13^, χ^2^ = 0.9, *df* = 14, p = 0.34). We found no difference in the direction of brood movement along trails between non-foraging and foraging nests (AoD^14^, χ^2^ = 0.09, *df* = 1, p = 0.79). There is also significantly less movement of brood on trails between non-foraging and foraging nests than between pairs of foraging nests (AoD^15^, χ^2^ = 372, *df* = 15, p<0.001). If larvae are considered separately there is still no significant difference in direction of movement along trails between non-foraging and foraging nests (AoD^16^, χ^2^ = 1.81, *df* = 1, p = 0.178). There were not enough pupa carrying journeys observed to test separately. Non-foraging nests do not have a higher protein-related activity than expected for their size (see above: non-foraging nests as hunting and scavenging specialists).

#### Discussion

There is no evidence to suggest that non-foraging nests are used to speed the development of brood; they are neither found in more insolated areas, nor do they have more brood related activity associated with them, than foraging nests.

We found that there is no difference in canopy cover over non-foraging compared to foraging nests. This lack of difference may mean that non-foraging nests would not provide a different temperature regime to foraging nests and therefore not be useful as brood development chambers. It is, however, important to note that this result disagrees with other studies from the same site, both of which found significantly higher canopy cover over foraging nests than non-foraging nests [7,17]. Nest size is another confounding factor when investigating the potential advantages of non-foraging nests as brood development chambers: larger nests tend to be found in areas of higher canopy cover, and are likely to be better at metabolic heat production, than smaller nests [17]. Other untested factors such as undergrowth, aspect, altitude are all also likely to have important implications for the nest temperature [17]. More studies, in different environments, may be necessary to find the complete relationship between foraging status and canopy cover, and the relationships between insolation, internal temperature and brood development. It is clear that the relationship is not consistent either within or between colonies, and there is some overlap between the canopy covers of non-foraging and foraging nests.

However, even if the non-foraging nests were in areas of different canopy cover it would not necessarily imply that they were involved in brood development. The absence of increased brood exchange along trails between non-foraging nests and foraging nests compared to those between pairs of foraging nests, and the absence of directional movement of brood along trails between non-foraging and foraging nests, strongly suggests that non-foraging nests are not being used as specialised brood development chambers, at least not on the short timescale examined in the study. The absence of greater-than-expected protein collection behaviours also suggest that non-foraging nests do not contain higher amounts of brood than foraging nests.

The fact that non-foraging nests are not brood development specialists may be unsurprising. Moving brood between nests is likely to be risky due to factors such as the risk of desiccation, predation or being damaged during the journey. Given the importance of brood to ant colonies, the risks to brood during transportation may mean that even if there were a marginally faster development time in another nest, the risks may still be too high to make mass brood transportation a beneficial strategy. Similarly, as non-foraging nests are smaller, and more likely to be abandoned, than foraging nests it may be better to maintain the brood in the larger, safer nests.

### (4) Non-foraging nests as seasonal foragers

#### Predictions

Nests which appear to be non-foraging could simply be foraging at other times of the year: we used repeated remapping of the same colonies over the course of three years to assess if this is the case. The remapping of the colonies occurred yearly in late-spring and late-summer; we expect any seasonal foraging effects to be observable in these two, very different, periods in the annual colony life-cycle. Different aphid species are often present on different tree species. Aphids have complex life-cycles which can result in rapid population increases, blooms, at certain points in the annual cycle. These blooms may occur at different points in the life-cycle of different aphid species on different trees. Under the hypothesis that non-foraging nests forage to different tree species to take advantage of seasonal aphid blooms we predict that non-foraging nests will be closer to different species of tree than the species of tree closest to foraging nests.

#### Results

Of the 66 nests present for all three years of the study only two consistently changed role between spring and summer. One switched from non-foraging in spring to foraging in late-summer (and foraged to Scots pine, *Pinus sylvestris*), whereas the other switched from foraging in spring to non-foraging in late-summer (and foraged to larch, *Larix* spp.). The nearest tree to a particular nest can be inferred from the colony maps. The species of these nearest trees can then be assessed. The species of tree nearest to non-foraging nests are not significantly different from the species of tree nearest to foraging nests (AoD^17^ χ^2^ = 4.8, *df* = 4, p = 0.31).

#### Discussion

There are very few nests which show a seasonal switch between foraging and non-foraging. The low numbers of seasonally foraging nests suggests that seasonal foraging is not an important role of non-foraging nests in polydomous wood ant colonies. The lack of relationship between the species of the nearest tree and foraging status of the nests suggests that nests are unlikely to be specialising on aphids blooming on different species of tree at different times of year. Though, it is important to note we only had two time-points over the course of an entire foraging season, we could be missing more rapid changes in food availability on different trees. Previous studies have shown that red wood ants show a high degree of route and site fidelity when foraging, even to the extent of following the same routes after the winter quiescence [5,37]. This consistency to a particular route may suggest that the same foraging trees are providing honeydew for long-periods of time. If there is little seasonal variation in food availability in different trees at this site it may explain why there seems to be little seasonal variation in where wood ant colonies forage.

### (5) Non-foraging nests as a selfish strategy

#### Predictions

Rather than having a specific role, non-foraging nests may simply be acting selfishly, exploiting the foraging effort of the rest of the colony. For this selfish strategy to be maintained, the non-foraging nests must have a high degree of reproductive isolation from the other nests of the colony, allowing a distinct lineage of queens and workers to develop. This distinct lineage of queens and workers could then increase their fitness at the expense of the rest of the colony. For a nest to have a distinct lineage of queens and workers there would need to be little brood exchange between non-foraging nests and the rest of the colony. Similarly, as carried workers may not be able to navigate back to their home nest [38], we predict that a selfish non-foraging nest would exchange few workers with the rest of the colony.

#### Results

There is significantly less movement of brood along trails between non-foraging and foraging nests than between pairs of foraging nests (0.47 ±0.15% and 2.3 ±0.72% of journeys respectively; AoD^14^, χ^2^ = 0.09, *df* = 1, p = 0.79). However, there is still some movement of brood; on average 7.6 ± 3.1 brood items are transported within a 30 minute observation.

**Fig 5:**
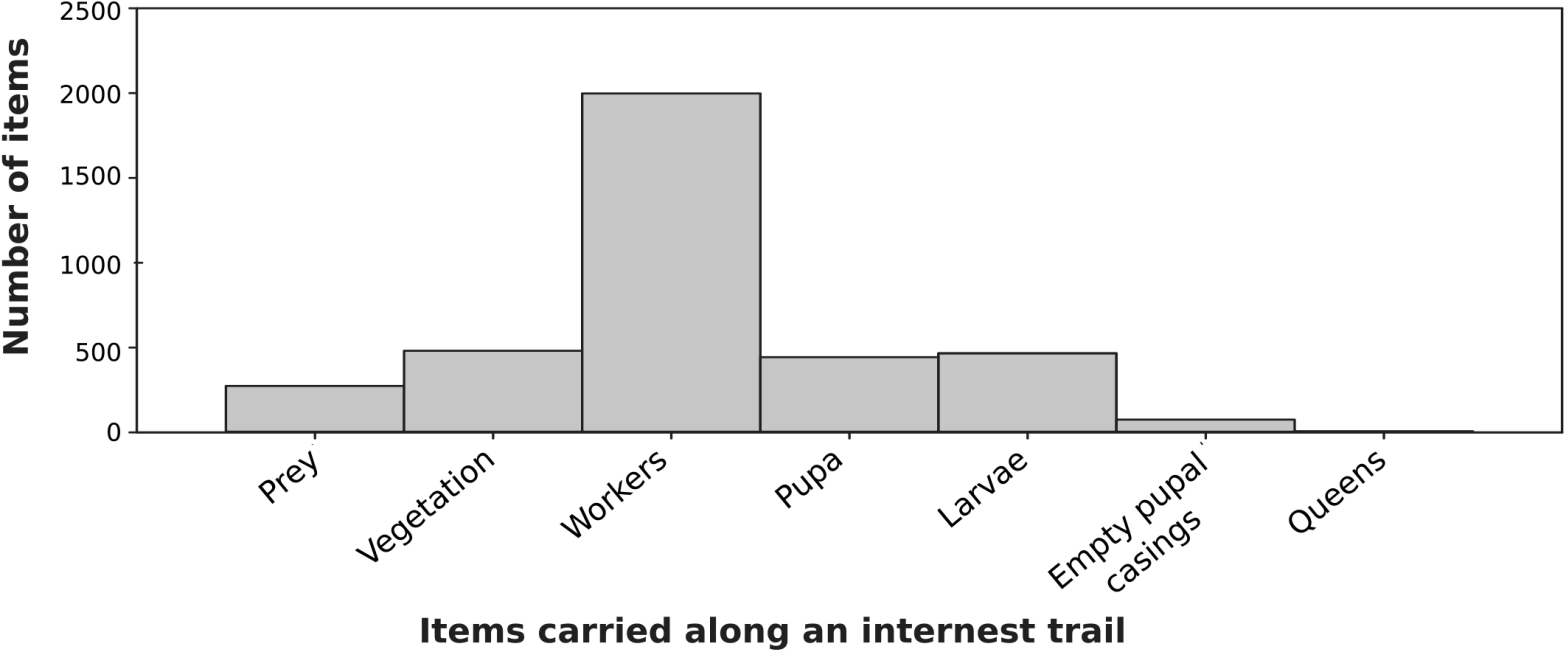
Items being carried between nests (dataset 2). The Fig shows the total number of items, summed across all 10 trials.

Workers were the most commonly observed item being carried between nests (Fig 5). Along trails between a non-foraging and a foraging nest there were mean of 34 ± 8.6 workers carried (summed for both trail directions) in a 30 minute observation.

#### Discussion

The impact of brood and worker movement between nests in polydomous *F*. *lugubris* colonies is difficult to assess. However the fact that there is any exchange at all indicates that the non-foraging nests are not completely reproductively isolated. Due to their high levels of polygyny, relatedness within polydomous red wood ant nests and colonies is general quite low [39]. This does not preclude more complex social and genetic structure within this generally low-relatedness social environment (e.g. [40,41]). The existence of brood and worker exchange between non-foraging nests and the rest of the colony suggests that if the non-foraging nests do have a distinct genetic lineage it is not a case of a simple strategy. This does not preclude non-foraging nests being part of a more complex intra-colony competition system, for example worker exchange could be a form of slave-making, but the relationship with the rest of the colony does not appear to be that of simply selfishly stealing resources. To understand the impact of the observed movement of brood and workers, and to understand the effect this has on intra-colony and intra-nest relatedness, it will be necessary to collect detailed genetic information over a long timescale.

### General Discussion

In this study we found that non-foraging nests in polydomous *F*. *lugubris* colonies appear to be part of the process of colony expansion. Our results do not suggest a specialised role, such as hunting for arthropod prey or brood development, for non-foraging nests. The process of colony expansion in polydomous wood ant colonies appears to be based on new nests being founded in random (with respect to food) locations. Of these newly founded nests, those which begin foraging are retained whereas those which do not are abandoned. This is a colony expansion strategy which is not available to monodomous colonies. For a monodomous colony, nest foundation must either be successful or the colony fails, whereas for a polydomous colony nest foundation can fail without long term fitness consequences for the colony. Non-foraging nests do not appear to have a specialised role within the colony. Rather than having a specialised role and providing a benefit to the entire colony, we found that non-foraging nests actually survive, in the long term, based on their own ability to acquire food (by beginning foraging). This agrees with other studies of polydomous red wood ants which have suggested a lack colony-level organisation [7] and a simple worker behaviours facilitating honeydew redistribution between nests of the polydomous colony [25]. Rather than a polydomous colony acting as cohesive whole, with shared survival and fitness prospects, the individual nests may survive based on their own ability to acquire resources, with little reference to the rest of the colony. Part of that acquisition of resources involves taking food from other nests in the colony [25] which is clearly a form of passive support so the nests are not entirely independent. However, nests within the polydomous colony appear to offer little active support to other nests.

The role of non-foraging nests as part of the colony expansion process also suggests an interesting dynamic within polydomous colonies between nest-level co-operation and nest-level selection. On one hand, non-foraging nests are founded and then, in effect, supported by the rest of the colony, providing very little benefit in return until they begin foraging: an example of nest-level co-operation. On the other hand, non-foraging nests which do not begin foraging are regularly abandoned, a strong form of nest-level selection. The dynamic between these two effects may be very important in determining a colony’s foraging success and the extent to which colonies’ expand. Both of these are likely to have important consequences for the long-term fitness of the colony.

## Supporting Information

S1 Table Details of the statistics used in the study. # refers to the superscript number in the text. The Dependent variable, fixed effects and random effects describe the GLMM used, all used a binomial error structure. In all tests errors were heteroscedastic and were not overdispersed. χ2, *df* and *P* describe the results of an analysis of deviance, which compares the model to a null model which lacks the variable of interest.
(DOCX)
