# Correction: The Role of Non-Foraging Nests in Polydomous Wood Ant Colonies

**DOI:** 10.1371/journal.pone.0143901

**Published:** 2015-11-23

**Authors:** The PLOS ONE Staff

Fig 2 is incorrect. The colors were inverted. The authors have provided a corrected version here. The publisher apologizes for the error.

**Fig 2:**
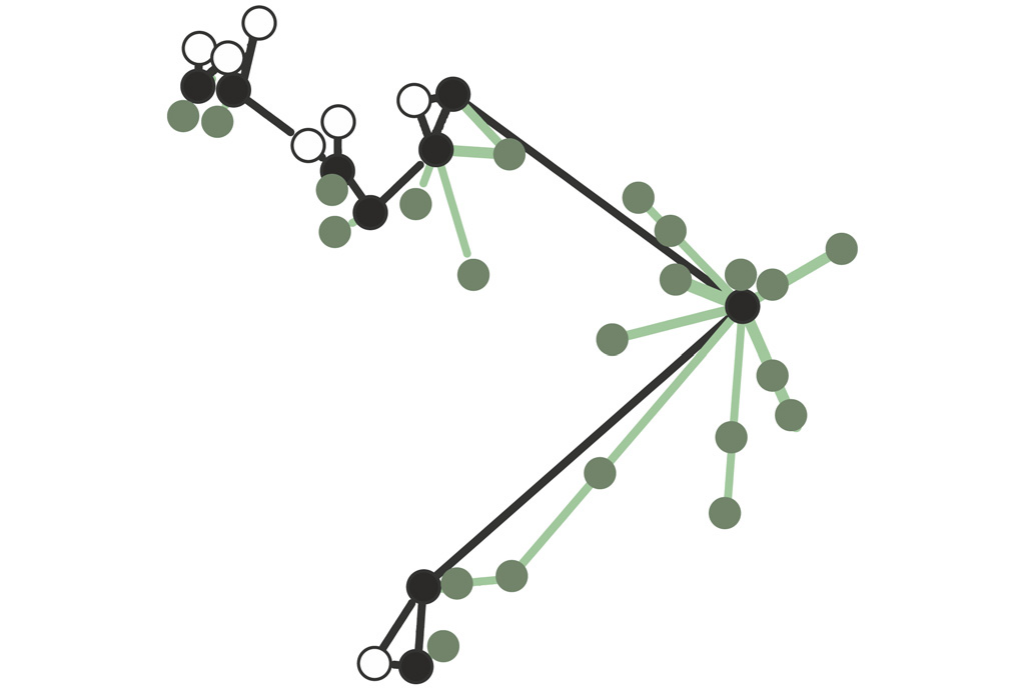
A polydomous *Formica lugubris* colony. Black circles represent foraging nests; open circles show non-foraging nests. Green circles are trees. Black lines are internest trails and green lines are foraging trails. Any nest without foraging trails leading to a tree is defined as non-foraging.
