# Supplementary material for: The Role of Non-Foraging Nests in Polydomous Wood Ant Colonies
Source: PLoS One. 2015 Oct 14;10(10):e0138321. doi: 10.1371/journal.pone.0138321 (PMC4605684; doi:10.1371/journal.pone.0138321)
Supplement: S1 Table — # refers to the superscript number in the text. The Dependent variable, fixed effects and random effects describe the GLMM used, all used a binomial error structure. In all tests errors were heteroscedastic and were not overdispersed. χ2, df and P describe the results of an analysis of deviance, which compares the model to a null model which lacks the variable of interest. (DOCX) [file pone.0138321.s001.docx]

| **#** | **Dependent Variable** | **Fixed Effect(s)** | **Random Effect(s)** | **χ^2^** | ***df*** | ***P*** |
| --- | --- | --- | --- | --- | --- | --- |
| 1 | Survival | F/nF | Colony  Year | 5.63 | 1 | 0.01 |
| 2 | Survival | Nest size | Colony  Year | 56.1 | 1 | <0.001 |
| 3 | Survial | F/nF  Nest size | Colony  Year | 5.64 | 1 | 0.03 |
| 4 | Survial | F/nF  Nest size | Colony  Year |  |  |  |
| 5 | Changed/Unchanged | F.nF | Colony  Nest ID | 13.7 | 2 | <0.001 |
| 6 | Changed/Unchanged | Relative distance to the nearest | Colony  Nest ID | 4.21 | 1 | 0.04 |
| 7 | Changed/Unchanged | Survival | Colony  Nest ID | 9.5 | 1 | 0.002 |
| 8 | Relative number of extra-nest workers | F/nF | Colony\|day  Temperature | 19.19 | 1 | <0.001 |
| 9 | Relative number of extra-nest workers | F/nF  Colony | Colony\|day  Temperature | 54.7 | 14 | <0.001 |
| 10 | Prey towards nF nest | Prey towards F nest | Colony  Temperature | 0.043 | 1 | 0.84 |
| 11 | Prey bearing journeys (prop.) | Trail type  Colony | Colony\|day | 128 | 15 | <0.001 |
| 12 | F/nF | Canopy Cover | Colony | 0.9 | 1 | 0.34 |
| 13 | Brood towards nF nest | Brood towards F nest | Colony  Temperature | 0.09 | 1 | 0.80 |
| 14 | Brood bearing journeys (prop.) | Trail type  Colony | Colony\|day | 371 | 15 | <0.001 |
